# Substance of Lectures on Gold

**Published:** 1878-10

**Authors:** 


### *Substance of Lectures on Gold.*—Continued.

BY PROF. HODGKIN.

The temperature at which pure gold is rendered fluid is quite high, but easily attained by our ordinary appliances. The mass in the crucible, to which our attention has been called and which we are submitting to the process of "dry refining," will probably fuse at a considerably lower temperature, as the almost universal law is that alloys have a lower melting point than the mean of their constituents. A little above red heat will probably serve to keep fluid the mass we are operating upon.

We bear in mind that our object is to purify the gold from its contaminations, and for that purpose we have reduced the whole mass to a fluid, (molten) condition. Only its surface can be acted upon by the agents used in refining, and so it is necessary to stir it up, using for this purpose the porcelain rod before spoken of.

*Reagents* are defined usually as agents used to detect the presence of substances. We use the word in a somewhat different or extended sense, as our reagents are used to eliminate from the gold its impurities.

The chemistry of their action is simple. Certain substances are brought into contact with these highly heated metals; the heat decomposes these complex bodies into their ultimate elements; such of these ultimate elements as have affinity for the metals in question unite with them, the others escaping.

To make this a little more full and explicit. The chemicals selected as efficient for our purpose must contain only such elements as will combine with the baser metals, but not with the gold, and they must further contain such elements as will in some way make such a combination with these base metals, as can be removed from the crucible. Several substances may be used for this purpose, possessing these characteristics. The essential feature of all is combination with oxygen or chlorine; the former is most commonly used. Nitrate of Potash (saltpetre) a salt consisting of nitric acid and potassa, is one of the simplest of our reagents, and its action may be easily understood.

The heat decomposes the salt, which is resolved into its ultimate elements, the nitrogen and potassium are volatile and fly off, having no affinity for the metals in whose presance they are liberated. The oxygen in its newly set-free condition is eager in its search for affinities, and greedily takes up with whatever presents itself of an oxydizable form. Oxygen in its *nascent state*, as it is called, or condition of being born, has an energy of combination not seen under other circumstances, and it readily oxydizes the baser metals, which in their molten condition, are exposed to its action.

Small pieces of saltpetre are thrown into the crucible from time to time and the contents brought into contact with it by stirring, and thus they are changed from the metallic form to that of compounds with oxygen. But we find that the tendency is strong on the part of these oxides to relapse to their metallic state, and recombine or alloy with the metals we are endeavoring to purify. So a second step is necessary—that of fixing them in their newly combined state. For this purpose borax (bi-borate of soda) is useful, as it strongly combines with these oxides of the metals, and form with them a sort of a glass of borax, which compound has no tendency to reduction, and which, floating on the surface of the metals, is easily removed.

Such is the simple process called *Dry Refining*, very old, and only partially satisfactory. It will, if carefully conducted, remove the lower metals, but it leaves in the alloyed mass untouched any silver or platinum which may be present there. For much of our work this is not of importance, as approximate purity will answer for our plates perhaps; and the process is exceedingly useful as a preliminary step to *Wet Refining*, of which we shall come to speak presently.

As adjuncts to this process several modifications are made. I have mentioned *chlorine* as one of the reagents. A stream of this gas injected into the crucible upon the surface of the molten metal, will soften and toughen it. The bi-chloride of mercury (corrosive sublimate,) thrown into the crucible, seems to answer well. Its action is not well understood. A piece of gold unmalleable by reason of impurity, if heated and thrown into dilute muriatic acid, seems to be softened, and rendered more manageable.

To sum up then, the principles involved in refining by the dry process are (1) heat, and (2) oxydation of impurities and removal of these oxides by the use of a flux which combines with these and renders their reduction to the metallic form difficult. As I have stated, it is proximate only, and no further purity can be expected than that of freedom from oxydizable metals. Those which are not capable of being thus treated, must be removed by what is known as the "wend process," to which I invite your attention.

---

### ARTICLE V.

### *Virginia State Dental Association.*

